# Research progress of short stature and advanced bone age, early-onset osteoarthritis and osteochondritis dissecans (SSOAOD) caused by acan gene mutation

**DOI:** 10.3389/fendo.2026.1809260

**Published:** 2026-05-21

**Authors:** Yunchong Chen, Su Wu, Wei Gu

**Affiliations:** Department of Endocrinology, Children’s Hospital of Nanjing Medical University, Nanjing, China

**Keywords:** ACAN gene, advanced bone age, growth hormone therapy, short stature, osteoarthritis

## Abstract

**Introduction:**

Heterozygous mutations in the *ACAN* gene are a key genetic cause of non-syndromic familial short stature. The associated disease spectrum includes short stature, advanced bone age, early-onset osteoarthritis, and osteochondritis dissecans (SSOAOD). The clinical phenotype is highly heterogeneous, ranging from isolated short stature to severe skeletal developmental abnormalities, reflecting a continuous pathological process from growth plate dysfunction to premature joint degeneration.

**Methods:**

This summary synthesizes current clinical evidence and management approaches for ACAN-related short stature. Recombinant human growth hormone (rhGH) is the primary treatment used to improve height in affected children. Clinical management also integrates growth promotion, joint protection, genetic counseling, and screening of at-risk family members.

**Results:**

rhGH therapy provides moderate improvement in height, but its efficacy decreases with increasing age. Long-term safety of rhGH on joint health remains unclear. The disease spectrum illustrates progressive severity from short stature alone to significant skeletal and joint pathology.

**Discussion:**

Clinical care should adopt a multidisciplinary approach combining growth promotion, joint protection, and genetic counseling, including screening for at-risk family members. Future multicenter, long-term follow-up studies are needed to further clarify the molecular mechanisms of *ACAN* haploinsufficiency, assess the long-term articular effects of rhGH therapy, and establish early identification criteria, thereby providing a basis for developing targeted therapies.

## 1 Introduction

Short stature is one of the most common clinical concerns in pediatric endocrinology, and its etiology is complex. With the development of molecular genetics, a considerable number of cases previously classified as “idiopathic short stature (ISS)” have been confirmed to be caused by single-gene variants. Among them, heterozygous mutations in the ACAN gene (which encodes the Aggrecan protein) are important causes of concern following SHOX in recent years. SSOAOD, short for Short Stature and Advanced Bone Age, Early-Onset Osteoarthritis and Osteochondritis Dissecans, is the core disease spectrum associated with ACAN gene heterozygous mutations (1).

SSOAOD is an autosomal dominant genetic disease caused by heterozygous mutations in the *ACAN* gene, mainly manifested as non-syndromic short stature, and some patients have bone problems such as advanced bone age, premature growth cessation, early-onset osteoarthritis and/or osteochondritis dissecans. Its clinical features (including short stature, advanced bone age and joint lesions) are often hidden in childhood, and are easily missed or misdiagnosed as isolated short stature. However, the impact of the disease on patients runs through the entire lifespan. Early identification and diagnosis not only facilitate timely growth interventions, such as individualized recombinant human growth hormone (rhGH) therapy, but significantly contribute to the mitigation of severe adult-onset osteoarthritis risk and the enhancement of long-term joint function prognosis. The key to enhancing patient quality of life, therefore, lies in improving clinical recognition of SSOAOD and establishing early screening combined with long-term follow-up strategies. Although reports of *ACAN*-related SSOAOD cases are accumulating, systematic characterization of the disease spectrum, standardized early diagnostic criteria, and evidence-based assessment of the long-term risk-benefit profile of growth hormone (GH) therapy remain elusive, with no established management consensus in clinical practice (2, 3).

## 2 Biological functions of *ACAN* gene and Aggrecan protein

### 2.1 Structure of *ACAN* gene

*ACAN* gene, located on human chromosome 15q26.1, contains 19 exons and encodes Aggrecan composed of 2316 amino acids.

### 2.2 Structure and function of Aggrecan protein

**Figure 1:**
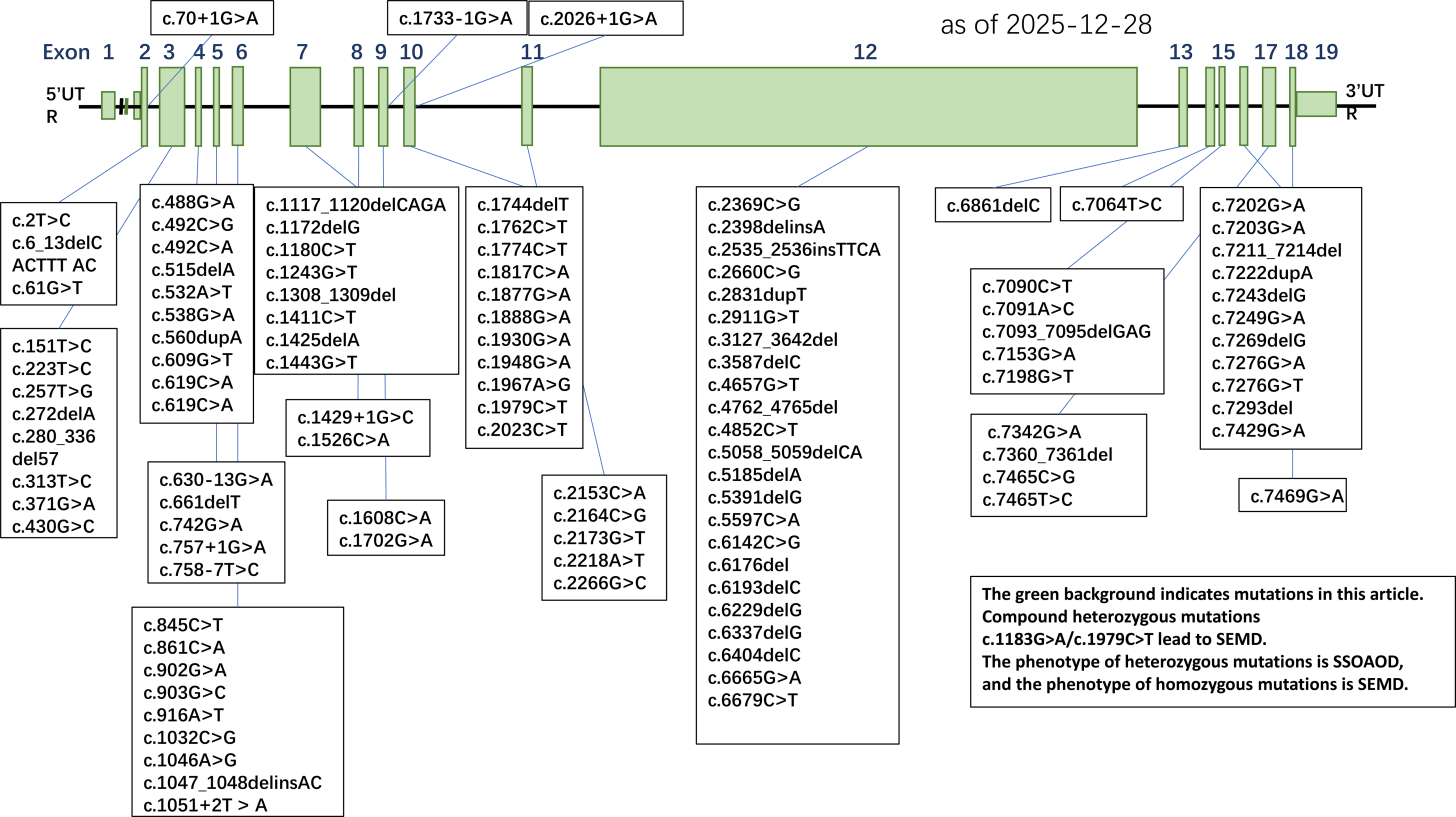
Summary of reported ACAN gene mutations.

Aggrecan (*ACAN*) is a proteoglycan that mainly exists in the extracellular matrix of cartilage. Its structure includes (i) an N-terminal signal peptide; (ii) two globular domains (G1 and G2); (iii) glycosaminoglycan (GAG)-attachment regions enriched with keratan sulfate (KS) and chondroitin sulfate (CS1, CS2); and (iv) a C-terminal G3 domain containing EGF-like, C-type lectin-like, and complement regulatory protein (CRP)-like subdomains (4) (**Figure 1**). G1 domain can bind to hyaluronic acid, while G3 interacts with fibrillar extracellular matrix proteins through lectin domain (5). The overall structure endows cartilage with compressive strength by forming highly hydrated gel, maintains tissue structure in growth plate and articular cartilage, and regulates chondrocyte proliferation and differentiation, which is essential for bone development and joint function (4, 6). Consequently, *ACAN* constitutes a pivotal functional component of the cartilage matrix. Pathogenic variants in *ACAN* disrupt growth plate organization, impair load-bearing capacity, and dysregulate chondrocyte lineage determination, thereby establishing the core pathophysiology of SSOAOD.

## 3.Pathogenic mechanisms of SSOAOD

### 3.1 At the cellular level

The pathogenic mechanism of SSOAOD forms a coherent pathological pathway at the cellular level: *ACAN* haploinsufficiency first leads to the reduction of extracellular matrix (ECM) synthesis and structural abnormalities (4), and then affects the survival and function of chondrocytes by disrupting the microenvironment home ostasis and triggering endoplasmic reticulum stress (7). At the same time, the disorder of transcriptional regulation (such as the decrease of SOX9 expression) (8) and abnormal signal transduction exacerbated the growth plate dysfunction; the key link is that the expression of CAMK1D is up-regulated, which then inhibits the PI3K/Akt signaling pathway, crucial for chondrocyte hypertrophy and differentiation (9). Finally, it leads to the impaired chondrocyte hypertrophy and linear growth disorder (10). It is worth noting that the core role of the above Akt signaling pathway provides a molecular basis for the treatment of growth hormone (GH) - GH can activate this pathway by increasing the level of IGF-1, so as to compensate for the signal suppression caused by *ACAN* deficiency to a certain extent, and provide a molecular explanation for the favorable response of children with heterozygous *ACAN* variant to GH treatment (9).

### 3.2 Evidence from animal models

Studies using mouse models of ACAN haploinsufficiency have revealed that the pathogenic mechanism of SSOAOD presents a continuous pathological process from structural defects to signaling dysregulation: *ACAN* haploinsufficiency first leads to a significant reduction in the extracellular matrix (ECM) of the growth plate, and then causes the height of the whole growth plate and its partitions (resting zone, proliferation zone, hypertrophic zone) to decline, directly destroying the normal spatial structure and microenvironment of chondrocytes (11). Secondly, the loss and structural abnormality of ECM further impaired the differentiation process of chondrocytes, which was manifested as the impaired differentiation of hypertrophic chondrocytes, accompanied by the down-regulation of the expression of key cartilage related genes (such as COL10A1, IHH, PTCH1, FGFR3) (6, 11). More importantly, the animal model revealed that ACAN deficiency not only caused structural “matrix reduction”, but also aggravated “hypertrophic impairment” by disrupting key intracellular signal transduction (such as decreased expression of SOX9 and inhibition of PI3K/Akt signaling pathway) (9, 10). These changes together hinder the hypertrophy process of chondrocytes, leading to growth plate failure, and ultimately resulting in linear growth disorder (10, 11).

## 4 Conceptual evolution and clinical manifestations of SSOAOD

### 4.1 Definition of SSOAOD

SSOAOD was first proposed by Nilsson et al. In 2014 to describe a group of non-syndromic short stature syndrome in individuals with heterozygous mutations in *ACAN* gene (12). Its characteristics include short stature, advanced bone age, premature growth cessation, and in some cases early-onset osteoarthritis or osteochondritis dissecans. With the accumulation of more cases, it was found that not all patients have advanced bone age, and the phenotype is heterogeneous (2). Therefore, the concept of SSOAOD has gradually evolved from focusing on “advanced bone age” to confronting a core phenotypic entity included in the *ACAN*-related disease spectrum, reflecting the deepening understanding of the disease (2, 13).

### 4.2 Clinical manifestations SSOAOD

#### 4.2.1 Short stature

At present, the reported SSOAOD patients are characterized by symmetrical short stature, which is mild before puberty, but the adult height is markedly reduced due to the premature cessation of pubertal growth. About 1.4% of non-syndromic short stature and 2.5% of familial short stature are associated with this gene. A total of 119 previously reported clinical phenotypes of SSOAOD children were summarized, of which 96 cases had a height SDS of<-2, and the proportion of short stature was 80.7%. The age distribution of these short stature patients was mainly concentrated under the age of 10 (accounting for 85.4% of the total number of short stature), especially between 3 and 9 years of age (accounting for 70.8% of the total number of short stature), suggesting that SSOAOD related short stature has been significantly manifested from preschool to early school age. Some patients were still short after the age of 10. At present, there are few reports about the final height of children. Final height data were available for only four patients. The average SDS value of final height was -3.47, all lower than -2 (100%), and the lowest was -4.37. Therefore, *ACAN*-related short stature not only occurs early in childhood, but also may continue to affect adults. The final height is substantially reduced, highlighting the importance of early diagnosis, long-term management and appropriate height intervention.

#### 4.2.2 Advanced bone age

Advanced bone age has previously been considered to be the characteristic feature of patients with short stature caused by *ACAN* mutation (2), and it is believed that short stature is related to advanced bone age and premature cessation of growth (2, 3, 12, 14). In our recorded patient data (a total of 40 cases), there were 19 cases with advanced bone age, accounting for 47.5%. The age distribution of these cases with advanced bone age shows a distinct pattern: it is concentrated mainly in the age range of 5 to 12 years old, especially in the age range of 7 to 10 years old, suggesting that this period is a window of bone age acceleration. In the clinical context, these children often associate early puberty or rapid progress. The advanced bone age further compresses the growth potential and significantly increases the risk of final adult height impairment, which has become one of the key factors restricting height growth and affecting the prognosis of terminal height. The changes of bone age should be closely monitored in clinical management, and the process of the pubertal development should be timely evaluated and intervened.

#### 4.2.3 Osteoarthritis

*ACAN* mutations not only affect the growth plate cartilage, but also the articular cartilage. Osteochondritis Dissecans (OCD) is characterized by the separation of articular cartilage and subchondral bone fragments from the joint surface. So far, about 1/3 of the patients reported in the literature exhibit OCD and early-onset OA phenotype (15). In the collected clinical phenotypes, only 4 patients or their relatives clearly reported osteoarthritis or related joint lesions. However, considering that the patient cohort is mainly children and adolescents, it is not surprising that typical osteoarthritis, which is more common in adults, is rarely observed in this group. The cases that have been found are teenagers or adult relatives, consistent with the age-related progression of the disease. In addition to two cases of typical osteoarthritis, there was also two cases of osteochondritis dissecans, which are closely related to the integrity of cartilage in adolescents, suggesting that the abnormal quality of articular cartilage caused by SSOAOD is an important pathological basis for the occurrence of osteoarthritis in the future.

#### 4.2.4 Other clinical manifestations

Other manifestations include midface hypoplasia, frontal bossing, flat nasal bridge, ong philtrum, short fingers, wide thumb, scoliosis, early-onset lumbar disc herniation and short neck, which collectively reflect the extensive role of *ACAN* in the development of cartilage and skeletal system (12, 13, 16). Therefore, SSOAOD is currently more likely to be regarded as a representative clinical phenotype in the spectrum of *ACAN*-related diseases, rather than an independent syndrome for which advanced bone age is considered a required diagnostic criterion.

## 5 SSOAOD variation types and genotype-phenotype correlation

### 5.1 SSOAOD variation types

Pathogenic variants in *ACAN* associated with SSOAOD exhibit significant mutational heterogeneity, including missense mutations, nonsense mutations, frameshift mutations, splice-site mutations and small insertions/deletions. These mutations are distributed across different exons of the gene, affecting multiple functional domains of *ACAN* protein, especially the G3 domain, resulting in abnormal protein function, which leads to growth plate cartilage development disorders and clinical phenotypic heterogeneity (9, 17–40). At present, a total of 114 mutations are included. Mutation types mainly include missense mutation, nonsense mutation, frameshift mutation, splice-site mutation, deletion (15, 41, 42),start loss and unknown type (unclassified). Among them, missense mutations (38, accounting for 33.6%), nonsense mutations (35, accounting for 31.0%) and frameshift mutations (30, accounting for 26.5%) were the main mutations, accounting for 91.1% of the total mutations. These mutations were widely distributed in various functional domains of protein, especially in G1 domain (G1-A, G1-B, G1-B’, a total of 36), C-terminal domain (CS/CS1/CS2, a total of 23) and G3 domain (a total of 22), accounting for 71.7% of the total number of mutations. Other mutations were scattered in the functional regions of signal peptide, immunoglobulin-like domain (IgD), G2-B/G2-B’ and keratan sulfate attachment region (KS).

### 5.2 SSOAOD genotype-phenotype correlation

At present, the genotype–phenotype correlation of SSOAOD caused by *ACAN* gene mutation is not clear. Although more than 59 families and 114 cases of pathogenic mutations have been reported, including missense, nonsense, frameshift, splice-site and other mutation types, patients in the same family also show a high degree of phenotypic heterogeneity (43). Some patients show advanced bone age, premature growth cessation, possibly accompanied by early-onset osteoarthritis or osteochondritis dissecans, while others may only have mild short stature, suggesting that the phenotype is modified by other genetic or environmental factors. However, the specific mechanism remains to be elucidated. It is necessary to further elaborate the mechanism of phenotypic heterogeneity in detail. For example, it is necessary to clarify whether advanced bone age is associated with specific mutation types, and analyze the correlation between different mutation types and bone age abnormalities combined with clinical cases to provide a basis for clinical diagnosis (15, 41, 42). Currently, emerging genotype-phenotype correlations are beginning to be characterized.

#### 5.2.1 Functional domain differences

##### 5.2.1.1 Nonsense/frameshift mutations in the G1/G2 domain are mainly characterized by isolated short stature

The reported nonsense/frameshift mutations in the G1/G2 domain (c.272delA, c.5391delG, etc.) lead to haploinsufficiency, with the phenotype of isolated short stature (44), and with fewer joint manifestations. The main mechanism is that mutation leads to premature termination codon (PTC), which leads to nonsense mediated mRNA degradation and protein expression decline, and finally shows an effect on growth plate cartilage, causing short stature. Among the patients in our study, the average height SDS of patients with G1 domain mutation (n=23) was -3.2, which met the criteria for short stature, and 47.8% were accompanied by typical facial features (such as frontal bossing, flat nasal bridge) or short finger/broad thumb and other skeletal manifestations, suggesting that this region may play an important role in the formation of early cartilaginous template and limb development.

##### 5.2.1.2 G3 missense mutations in the lectin region are more likely to be associated with OCD/OA

At present, many families with familial osteochondritis dissecans have been reported in the literature, including multiple mutations (p.C2386S, p.L2417P, p.D2453V, p.V2455M, p.L2355P, etc.) (1, 12, 45) which are located in the core region of the C-type lectin repeat region of the G3 domain of *ACAN* gene (1, 45), and their clinical phenotypes are highly consistent. It can be inferred that the missense variation of the C-type lectin region of the G3 domain of *ACAN* gene is a shared molecular mechanism underlying the specific phenotype of familial OCD with short stature and early-onset osteoarthritis by disrupting protein folding, secretion and extracellular matrix interactions (1, 22, 45).

#### 5.2.2 Differences in mutation types

##### 5.2.2.1 Mutation type alone is insufficient for predicting the severity of phenotype, suggesting the presence of modifier factors

Published studies indicate that the mutation types (missense, truncating mutations, Nonsense, frameshift, etc.) are not clearly related to the phenotypic severity or the occurrence of joint disease (43). However, there are also reports of a new truncated variant of the 5 ‘end of *ACAN* gene, whose phenotype is characterized by severe non-syndromic short stature, without advanced bone age or significant joint disease (47).

## 6 Advances in treatment and management

### 6.1 GH therapy

At present, GH therapy is still the main strategy for the treatment of SSOAOD caused by *ACAN* gene mutation. Most of the previous literatures reported that GH treatment was effective (2). A small number of literature indicated that GH treatment only accelerated growth (43). The consensus of most articles is that the combined treatment of GnRHa can effectively inhibit the progression of bone age (12, 43) and ultimately increase the final height by 4–6 cm. However, there is a lack of long-term follow-up data and summary, and the final height of some children is still significantly below the normal range (12, 45).

Accordingly, we searched PubMed and CNKI (as of December 30, 2025) for articles reporting ACAN variants associated with short stature, and collected and summarized the relevant clinical data of patients, especially about the height standard deviation score (SDS), phenotypic characteristics and genetic characteristics. The patient recruitment was carried out through systematic literature retrieval, and the inclusion criteria were children with clear ACAN pathogenic mutations and complete height and clinical phenotype data, excluding those with other genetic diseases and severe organ diseases. A total of 152 children were included, including 65 children with growth hormone treatment data. This part is a secondary descriptive analysis based on published literature. See **Table 1** for references of non-systematic meta-analysis (no weighting and no bias assessment).

**Table 1:** Summary of genotypes related to SSOAOD.

No.	Nucleotide change	Homozygous / Heterozygous	Protein change	Exon / Intron	Protein domain	Mutation type	De novo	Disease	References
1	c.1_2delAT	Het	?	1	G1	Missense	N	SSOAOD	(19)
2	c.1_9del	Het	p.Met1?	–	–	–	N	SSOAOD	(46)
3	c.2T>C	Het	–	E2	–	start loss	N	SSOAOD	(25)
4	c.6_13delCACTTTAC	Het	p.Thr3Leufs*21	E2	Signal peptide	Frameshift	N	SSOAOD	(47)
5	c.61G>T	Het	p.Glu21*	E2	Signal peptide	Nonsense	N	SSOAOD	(2, 23)
6	c.70+1G>A	Het	–	I2	–	Splicing	N	SSOAOD	(26)
7	c.151T>C	Het	p.Cys51Gly	E3	G1-A	Missense	N	SSOAOD	(27)
8	c.223T>C	Het	p.Trp75Arg	E3	G1-A	Missense	N	SSOAOD	(2)
9	c.257T>G	Het	p.Leu86Arg	E3	G1-A	Missense	N	SSOAOD	(25)
10	c.272delA	Het	p.Arg93Alafs*41	E3	G1-A	Frameshift	N	SSOAOD	(2, 12)
11	c.280_336del57	Het	p.Val94_Ile112del	E3	G1-A	?	N	SSOAOD	(28)
12	c.313T>C	Het	p.S105P	E3	G1-A	Missense	N	SSOAOD	(29)
13	c.371G>A	Het	p.Arg124His	E3	G1-A	Missense	N	SSOAOD	(23)
14	c.430G>C	Het	p.Ala144Pro	E3	G1-A	Missense	N	SSOAOD	(25)
15	c.488G>A	Het	p.Arg163His	E4	G1	Missense	N	SSOAOD	(19)
16	c.492C>G	Het	p.Tyr164*	E4	G1-B	Nonsense	N	SSOAOD	(2)
17	c.492C > A	Het	p.Tyr164*	E4	G1-B	Nonsense	N	SSOAOD	(26, 28, 30)
18	c.512C>T	Hom	p.Ala171Val	E4	G1-B	Nonsense	N	SSOAOD	(33)
19	c.515delA	Het	p.Gln172Argfs*59	E4	G1-B	Frameshift	N	SSOAOD	(27)
20	c.532A>T	Het	p.Asn178Tyr	E4	G1-B	Missense	N	SSOAOD	(2, 31)
21	c.538G>A	Het	p.Ala180Thr	E4	G1-B	Missense	N	SSOAOD	(26)
22	c.560dupA	Het	p.Leu188fs*13	E4	G1-B	Frameshift	N	SSOAOD	(32)
23	c.609G>T	Het	p.Trp203Cys	E4	G1-B	?	N	SSOAOD	(28)
24	c.619C > A	Het	p.Q207K	E4	G1-B	?	N	SSOAOD	(9)
25	c.630-13G>A	Het	p.Y211Ffs*24	E5	G1-B	Splicing	N	SSOAOD	(33)
26	c.661delT	Het	p.Tyr221Metfs*10	E5	G1-B	Frameshift	N	SSOAOD	(47)
27	c.742G>A	Het	p.Ala248Thr	E5	G1-B	Missense	N	SSOAOD	(23)
28	c.757+1G>A	Het	–	E5	G1-B	Splicing	N	SSOAOD	(34)
29	c.758-7T>C	Het	–	E5	G1-B	Splicing	N	SSOAOD	(29)
30	c.845C>T	Het	p.Thr282Ile	E6	G1-B’	Missense	N	SSOAOD	(25)
31	c.861C>A	Het	p.Tyr287*	E6	G1-B'	Nonsense	N	SSOAOD	(26)
32	c.902G>A	Het	p.Trp301*	E6	G1-B'	Nonsense	N	SSOAOD	(25)
33	c.903G>C	Het	p.Trp301Cys	E6	G1-B'	Missense	N	SSOAOD	(2, 23)
34	c.916A>T	Het	p.Ser306Cys	E6	G1-B'	Missense	N	SSOAOD	(2)
35	c.1032C>G	Het	p.Tyr344*	E6	G1-B'	Nonsense	N	SSOAOD	(26)
36	c.1046A>G	Het	p.Tyr349Cys	E6	G1-B'	Missense	N	SSOAOD	(35)
37	c.1047_1048delinsAC	Het	p.Tyr349*	E6	G1-B'	Nonsense	N	SSOAOD	(2)
38	c.1051+2T > A	Het	–	E6	G1-B'	Splicing	N	SSOAOD	(29)
39	c.1051+1G>A	Het	p.Val255_Glu352del	?	?			SSOAOD	(28)
40	c.1117_1120delCAGA	Het	p.Thr374*	E7	IGD	Nonsense	N	SSOAOD	(47)
41	c.1172delG	Het	p.Gly391Valfs*7	E7	IGD	Nonsense	N	SSOAOD	(28)
42	c.1180C>T	Het	p.Arg394*	E7	IGD	Nonsense	N	SSOAOD	(27)
43	c.1243G>T	Het	p.Glu415*	E7	IGD	Nonsense	N	SSOAOD	(2, 36)
44	c.1308_1309del	Het	p.Gly437Argfs*22	E7	IGD	Frameshift	N	SSOAOD	(9)
45	c.1411C>T	Het	p.Gln471*	E7	IGD	Nonsense	N	SSOAOD	(15)
46	c.1425delA	Het	p.Val478Serf s*	E7	G2-B	Frameshift	N	SSOAOD	(2)
47	c.1429+1G>C	Het	Val465Glyfs*13	E8	G2-8	Splicing	N	SSOAOD	(19, 26)
48	c.1443G>T	Het	p.Glu415*	E7	G2-B	Nonsense	N	SSOAOD	(2)
49	c.1526C>A	Het	p.Ser509*	E8	G2-B	Nonsense	N	SSOAOD	(2)
50	c.1608C>A	Het	p.Tyr536*	E9	G2-B	Nonsense	N	SSOAOD	(3, 23)
51	c.1702G>A	Het	p.Asp568Asn	E9	G2-B	Missense	N	SSOAOD	(27)
52	c.1733-1G>A	Het	–	I9	–	Splicing	N	SSOAOD	(15)
53	c.1744delT	Het	p.Phe582fs*69	E10	G2-B'	Frameshift	N	SSOAOD	(13)
54	c.1762C>T	Het	p.Gln588*	E10	G2-B’	nonsense	N	SSOAOD	(15)
55	c.1774C>T	Het	p.Gln592*	E10	G2-B'	Nonsense	N	SSOAOD	(27)
56	c.1817C>A	Het	p.Ala606Asp	E10	G2-B'	Missense	N	SSOAOD	(15, 26)
57	c.1877G>A	Het	p.W626	E10	G2-B'	Nonsense	N	SSOAOD	(37)
58	c.1888G > A	Het	p.G630S	E10	G2-B'	?	N	SSOAOD	(9)
59	c.1930G>A	Het	p.Gly644Ser	E10	G2-B'	Missense	N	SSOAOD	(23)
60	c.1948G>A	Het	p.Val650Met	E10	G2-B'	Missense	N	SSOAOD	(23)
61	c.1967A > G	Het	p.Y656C	E10	G2-B'	?	N	SSOAOD	(9)
62	c.1979C>T	Het	p.Thr660Met	E10	G2-B'	Missense	N	SSOAOD	(26, 35)
63	c.2023C>T	Het	p.Arg675*	E10	G2-B'	Nonsense	N	SSOAOD	(25, 28)
64	c.2026+1G>A	Het	–	I10	–	Splicing	N	SSOAOD	(2, 12)
65	c.2153C>A	Het	p.T718K	E11	KS	Missense	N	SSOAOD	(29)
66	c.2164C>G	Het	p.Pro722Ala	E11	KS	Missense	N	SSOAOD	(41)
67	c.2173G>T	Het	p.Glu725*	E11	KS	Nonsense	N	SSOAOD	(26)
68	c.2218A>T	Het	p.Thr740Ser	E11	KS	Missense	N	SSOAOD	(23)
69	c.2266G>C	Het	p.Gly756Arg	E11	KS	Missense	N	SSOAOD	(15)
70	c.2369C>G	Het	p.Ser790*	E12	KS	Nonsense	N	SSOAOD	(23)
71	c.2398delinsA	Het	p.Ser800Profs	E12	KS	Frameshift	N	SSOAOD	(26)
72	c.2535_2536insTTCA	Het	p.Pro846Phefs*9	E12	KS	Frameshift	NA	SSOAOD	(35)
73	c.2660C>G	Het	p.S887X	E12	KS	Nonsense	N	SSOAOD	(29)
74	c.2831dupT	Het	p.Glu945Argfs*484	E12	CS1	Frameshift	N	SSOAOD	(19)
75	c.2911G > T	Het	p.G971X	E12	CS1	Nonsense	N	SSOAOD	(29)
76	c.3127_3642del	Het	p.Ala1043_Ala1214del	E12	CS1	Missense	N	SSOAOD	(19)
77	c.3587delC	Het	p.Pro1196Leufs*3	E12	CS1	Frameshift	N	SSOAOD	(26)
78	c.4657G>T	Het	p.Glu1553*	E12	CS1	Nonsense	N	SSOAOD	(2)
79	c.4762_4765del	Het	p.Gly1588fs	E12	CS1	Frameshift	N	SSOAOD	(3)
80	c.4852C>T	Het	p.Gln1618*	E12	CS2	Nonsense	N	SSOAOD	(38)
81	c.5058_5059delCA	Het	p.Ile1686Metfs*13	E12	CS2	Frameshift	N	SSOAOD	(26)
82	c.5185delA	Het	p.lle1729Tyrfs*24	E12	CS	Frameshift	N	SSOAOD	(19)
83	c.5391delG	Het	p.Gly1797Glyfs*52	E12	CS2	Frameshift	N	SSOAOD	(2, 14)
84	c.5597C>A	Het	p.Ser1866*	E12	CS2	Nonsense	N	SSOAOD	(27)
85	c.6142C>G	Het	p.Pro2048Ala	E12	CS2	Missense	N	SSOAOD	(23)
86	c.6176del	Het	p.Pro2059Leufs*2	E12	CS2	Frameshift	N	SSOAOD	(25)
87	c.6193delC	Het	p.Gln2065Serfs*27	E12	CS2	Frameshift	N	SSOAOD	(39)
88	c.6229delG	Het	p.Asp2078Thrfs*14	E12	CS2	Nonsense	N	SSOAOD	(34)
89	c.6337delG	Het	p.Ala2113fs*17	E12	CS2	Frameshift	N	SSOAOD	(19)
90	c.6404delC	Het	p.Ala2135Aspfs	E12	CS2	Frameshift	N	SSOAOD	(38)
91	c.6665G > A	Het	p.Trp2222*	E12	CS2	Nonsense	N	SSOAOD	(26)
92	c.6679C>T		p.Gln2227*	E12	CS2	Nonsense	N	SSOAOD	
93	c.7064T>C	Het	p.Leu2355Pro	E14	G3	Missense	N	SSOAOD	(2, 12)
94	c.7090C>T	Het	p.Gln2364*	E15	G3	Nonsense	N	SSOAOD	(3)
95	c.7091A>C	Het	p.Gln2364Pro	E15	G3	Missense	N	SSOAOD	(48)
96	c.7093_7095delGAG	Het	p.Glu2365del	E15	G3	Deletion	NA	SSOAOD	(35)
97	c.7153G>A	Het	p.Glu2385Lys	E15	G3	Missense	N	SSOAOD	(2, 19)
98	c.7198G > T	Het	p.Glu2400*	E15	G3	Nonsense	N	SSOAOD	(26)
99	c.7202G>A	Het	p.Trp2401*	E16	G3	Nonsense	N	SSOAOD	(28)
100	c.7203G>A	Het	p.Trp2401*	E16	G3	Nonsense	N	SSOAOD	(2)
101	c.7211_7214del	Het	p.Asn2404Serfs*14	E16	G3	Frameshift	N	SSOAOD	(19)
102	c.7222dupA	Het	p.Asp2407fs	E16	G3	Frameshift	N	SSOAOD	(40)
103	c.7243delG	Het	p.D2415Tfs*4	E16	G3	Frameshift	N	SSOAOD	(29)
104	c.7249G>A	Het	p.Val2417Met	E16	G3	Missense	N	SSOAOD	(2)
105	c.7269delG	Het	p.Glu2424fs*5	E16	G3	Frameshift	N	SSOAOD	(23)
106	c.7276G>A	Het	p.Glu2426Lys	E16	G3	Missense	N	SSOAOD	(19, 23)
107	c.7276G>T	Het	p.Gly2426*	E16	G3	Nonsense	N	SSOAOD	(2, 23)
108	c.7293del	Het	p.Cys2432Alafs*139	E16	CLD	Frameshift	N	SSOAOD	(25)
109	c.7342G>A	Het	p.Gly2448Arg	E17	G3	Missense	NA	SSOAOD	(23)
110	c.7360_7361del	Het	p.E24 54fs	E17	G3	Frameshift	NA	SSOAOD	(37)
111	c.7429G>A	Het	p.Val2417Met	E16	G3	Missense	N	SSOAOD	(37)
112	c.7465C > G	Het	p.Arg2489Gly	E17	G3	Missense	N	SSOAOD	(26)
113	c.7465T>C	Het	p.Gln2364Pro	E17	G3	Missense	NA	SSOAOD	(48)
114	c.7469G>A	Het	p.Cys2490Tyr	E18	G3	Missense	N	SSOAOD	(15)

Among the 65 children treated with growth hormone, 24 were female and 41 were male. The minimum age was 2 years and 4 months, and the maximum age was 15 years and 1 month. 45 children showed short stature (< -2SD) before treatment. The median height SDS of all children at the beginning of GH treatment was -2.7 SD (-5.6 to -0.7), and the duration of treatment ranged from 1.5 months to 9 years (42 of them had treatment time ≥ 1 year). 15 children were treated with GnRHa. The average treatment SDS change value of all children was 0.59 SD (same for male and female), and the average SDS value of the first year of treatment was 0.68 SD (less than 1 year was annualized based on monthly data). After treatment, the median height SDS of children was -2 SD (-5 to 0.62), and only 30 patients still showed short stature (<-2SD). In our study, final height data were available for only four patients. In these cases, short stature persisted after treatment, but they became 4-5cm taller than the predicted adult height calculated at the first visit. This indicates that GH treatment has a moderate but associated growth-promoting effect on short stature caused by most ACAN gene mutations. This effect persists during the treatment period and has a better effect in the first year, and can change the final height of children to a certain extent (3, 46, 48).

After statistical analysis, the height was converted into sex- and age-adjusted SDS (CA) (**Table 2**). After confirming the data followed a normal distribution, Pearson correlation analysis was used to test the correlation between height SDS and age. Pearson correlation analysis revealed no significant correlation between the standard deviation score (SDS) of height before treatment and age (p = 0.30). The Pearson correlation coefficient between the average annual treatment SDS and age was-0.441 (p <0.001), while Pearson correlation coefficient between average annual SDS change and treatment duration was-0.457 (p <0.001). These findings suggest that treatment efficacy was inversely correlated with age or prolonged treatment duration.

**Table 2:** The mutation sites, clinical phenotypes, and treatment data of the ACAN gene.

Reported no	Nucleotide change	Hom/Het	Protein change	Exon/Intron	Domain	Mutation type	De novoc	Disease	Age	Sex	Height	Weight	Paternal height	Maternal height	SGA	Moderate facial dysmorphism	Short neck	Scoliosis	Brachydactyly	Other clinical features	IGF1	peakGH	BA before	BA-CA before	Age at GH introduction	Ht SDS before	GH duration (year)	Ht SDS after	Average growth SDS	Total growth SDS	GH dosage	Combination therapy duration(year; GnRHa, AI)	BA after	BA-CA after	Adult height SDS (AH)
1	c.1_2delAT	Het		1	G1	Missense	N	SSOAOD	8.5	0			175	145		0	0	0	0	Depressed nasal bridge, prominent forehead, cubitus valgus		27.4	10.8	2	8.8	-2.53	0.75	-1.45	1.44	1.08		0.75			
2	c.1_9del	Het	p.Met1?					SSOAOD																											
3	c.2T>C	Het		E2		start loss	N	SSOAOD	14.5	1	138.7	38			0	0	0	0	0	Hyperopia, broad nose, thin lips, long philtrum		8.58	13.5	-1	14.58	-4.1	0.4	-4.1	0	0	49.5ug/kg/day				
4	c.6_13delCACTTT AC	Het	p.Thr3Leufs*21	E2	Signal peptide	Frameshift	N	SSOAOD	7.58	1	104.8				0					Pectus excavatum		12.2	6.5	-1.08		-4.3	0.08								
5	c.61G>T	Het	p.Glu21*	E2	Signal peptide	Nonsense	N	SSOAOD	4.5	0					0	1		1	1	Macrocephaly			6	1.5		-3.5									
6	c.70+1G > A	Het		I2		Splicing	N	SSOAOD	14.25	1	132.2	45	158	168	0						167	7.332	11.5	/	14.25	-4.9	0.125	-5	/	-0.1	0.13IU/kg/d				
7	c.151T>G	Het	p.Cys51Gly	E3	G1-A	Missense	N	SSOAOD		0					0	0	0	0	0	Barrel chest, limited supination			+	+		-3.5									
8	c.223T>C	Het	p.Trp75Arg	E3	G1-A	Missense	N	SSOAOD	5.42	1															5.5	-2	1	-1.3	0.7	0.7	30-50 μg/kg/day				
9	c.223T>C	Het	p.Trp75Arg	E3	G1-A	Missense	N	SSOAOD	8.25	0															8.3	-1.9	1	-1.2	0.7	0.7	30-50 μg/kg/day				
10	c.280_336del57	Het	p.Val94_Ile112del	E3	G1-A	?	N	SSOAOD	3.33	1													5	1.67	3.33	-2.2	1	-1.13	1.07	1.07	50 μg/kg/day				
11	c.280_336del57	Het	p.Val94_Ile112del	E3	G1-A	?	N	SSOAOD	5	1													6	1	5	-1.12	1	-0.46	0.66	0.66	50 μg/kg/day				
12	c.257T>G	Het	p.Leu86Arg	E3	G1-A	Missense	N	SSOAOD	7.5	1	119.5	23.6			0							12.5	8.5	1		-1.5									
13	c.272delA	Het	p.Arg93Alafs*41	E3	G1-A	Frameshift	N	SSOAOD	8.42	1	111.3			145.9	1	1			1	Flat nasal bridge, mild mandibular prognathism			12	3.58	8.7	-3.3	2.6	-2.7	0.192307692	0.5	30-50 μg/kg/day	1			
14	c.272delA	Het	p.Arg93Alafs*41	E3	G1-A	Frameshift	N	SSOAOD	5.83	1	105.7			145.9		1			1	Flat nasal bridge, mandibular prognathism					6.3	-1.8	2.4	-1.6	0.083333333	0.2	30-50 μg/kg/day				
15	c.272delA	Het	p.Arg93Alafs*41	E3	G1-A	Frameshift	N	SSOAOD	27	0	145.9					1			1	Flat nasal bridge						-2.7									
16	c.313T > C	Het	p.S105P	E3	G1-A	Missense	N	SSOAOD	3.08	1	83.2	11.5			0	0	1	0	1	Prominent forehead, depressed nasal bridge	56		3.5	0.42	3.08	-3.65	0.67	-2.68	1.45	0.97	0.15 IU/kg/day				
17	c.371G>A	Het	p.Arg124His	E3	G1-A	Missense	N	SSOAOD	8	0					0			1	1	Broad nasal bridge, thin lips, prominent palate, hip pain			6	-2		-3.7									
18	c.430G>C	Het	p.Ala144Pro	E3	G1-A	Missense	N	SSOAOD	7.17	1	106.8	18.5			1	0	0	1	0	0		4.2	8.25	1.08	8.17	-3.4	0.8	-2.9	0.625	0.5	36.3ug/kg/day				
19	c.488G>A	Het	p.Arg163His	E4	G1	Missense	N	SSOAOD	11.6	1			172	153				1		Prominent forehead, depressed nasal bridge		13.3	10.7	0.9	11.83	-1.92	0.5	-1.94	0.46	0.23					
20	c.492C>G	Het	p.Tyr164*	E4	G1-B	Nonsense	N	SSOAOD																											
21	c.492C > A	Het	p.Tyr164*	E4	G1-B	Nonsense	N	SSOAOD	14	0	132.8	45.5	160	145	NA								16												
22	c.492C>A	Het	p.Tyr164*	E4	G1-B	Nonsense	N	SSOAOD	7.33	0													9.33	2	7.33	-3.11	1	-2.55	0.56	0.56	50 μg/kg/day				
23	c.512C>T	Hom	p.Ala171Val	E4	G1-B	Nonsense	N	SSOAOD	7	0	101	16	175	140	0		1		1		83.03	38.97	7	0											
24	c.512C>T	Hom	p.Ala171Val	E4	G1-B	Nonsense	N	SSOAOD	14	0	120				0		1		1																
25	c.515delA	Het	p.Gln172Argfs*59	E4	G1-B	Frameshift	N	SSOAOD		0					1	0	1	0	1	Frontal compression, barrel chest, limited supination			–	–		-3.6									
26	c.532A>T	Het	p.Asn178Tyr	E4	G1-B	Missense	N	SSOAOD	11.8	1			-3.9	0.9	1								13	1.2		-2									
27	c.538G > A	Het	p.Ala180Thr	E4	G1-B	Missense	N	SSOAOD	4.5	1	99.6	18	150	160							131	11.414	5.5	1	4.53	-2	1.17	-0.9	0.94017094	1.1	0.16IU/kg/d				
28	c.560dupA	Het	p.Leu188fs*13	E4	G1-B	Frameshift	N	SSOAOD																											
29	c.609G>T	Het	p.Trp203Cys	E4	G1-B	?	N	SSOAOD	6	1													7	1	6	–4.27	1	-3.89	0.38	0.38	50 μg/kg/day				
30	c.619C > A	Het	p.Q207K	E4	G1-B	?	N	SSOAOD	8.58	1	102	20	158	155							NA	13.1	5.5	-3.08	8.58	-5.6	5.83	-3.2	0.41	2.4		1			
31	c.630-13G > A	Het	p.Y211Ffs*24	E5	G1-B	Splicing	N	SSOAOD	2.92	0	86.4	12.75	176(0.55SDS)	148(-2.33SDS)		1			1	Mild hypertelorism, thumbs and great toes slightly broader than average	118	26.17	4.5	1.58											
32	c.661delT	Het	p.Tyr221Metfs*10	E5	G1-B	Frameshift	N	SSOAOD	12	1	130				0					Acanthosis nigricans		5.96	12			-2.96									
33	c.742G>A	Het	p.Ala248Thr	E5	G1-B	Missense	N	SSOAOD	14.5	1					0	0	0	0	1	Mild skeletal deformities, hip pain			14.5	0		-1.8									
34	c.742G>A	Het	p.Ala248Thr	E5	G1-B	Missense	N	SSOAOD										1		Early-onset osteoarthritis						-3									
35	c.757+1G>A	Het		E5	G1-B	Splicing	N	SSOAOD	4.17	1	94.1	15	175(−0.11 SD)	130(−5.16SD)	0			0					5.5	1.33		-2.49									
36	c.758-7T > C	Het		E5	G1-B	Splicing	N	SSOAOD	9.08	1	126.7	24			0	0	0	1	1		234		10.17	1.08		-1.61									
37	c.845C>T	Het	p.Thr282Ile	E6	G1-B'	Missense	N	SSOAOD	7.5	0	105.8				0			0	0	Hyperopia, broad nose, flat nasal bridge, pectus excavatum		23	7.5	0	8.17	-3.8	1.08	-3.2	0.56	0.6	33-39.6ug/kg/day				
38	c.861C > A	Het	p.Tyr287*	E6	G1-B'	Nonsense	N	SSOAOD	9.75	1	127.7	26.7	174	148	0						417		10	0.25		-1.77									
39	c.902G>A	Het	p.Trp301*	E6	G1-B'	Nonsense	N	SSOAOD	6.25	1	106	19			0			0	0				6.42	0.17	6.25	-2.9	0.58	-2.4	0.86	0.5	33ug/kg/day				
40	c.903G>C	Het	p.Trp301Cys	E6	G1-B'	Missense	N	SSOAOD	7	0					0	1	0	0	1	Depressed nasal bridge, slender femur, knee joint defect			7	0		-3.5									
41	c.916A>T	Het	p.Ser306Cys	E6	G1-B'	Missense	N	SSOAOD																											
42	c.1032C > G	Het	p.Tyr344*	E6	G1-B'	Nonsense	N	SSOAOD	11.17	1	126.9	33	150	156	NA								11	-0.17											
43	c.1046A>G	Het	p.Tyr349Cys	E6	G1-B'	Missense	NA	SSOAOD	6.67	0					0							17.2	6.1	-0.7		-3.06									
44	c.1047_1048delinsAC	Het	p.Tyr349*	E6	G1-B'	Nonsense	N	SSOAOD																											
45	c.1051+2T > A	Het		E6	G1-B'	Splicing	N	SSOAOD	3	1	84	10			0	1	1	0	1	Flat nasal bridge	37		2.5	-0.5	3	-3.46	0.5	-3.13	0.66	0.33	0.15 IU/kg/day				
46	c.1051 + 1G>A	Het	p.Val255_Glu352del						8.08	0													10	1.92		-2.99	1	-2.41	0.58	0.58					
47	c.1117_1120delCAGA	Het	p.Thr374*	E7	IGD	Nonsense	N	SSOAOD	13.25	1	133.4											5.94	13.5			-3.54									
48	c.1172delG	Het	p.Gly391Valfs*7	E7	IGD	Nonsense	N	SSOAOD	2.33	0													2.42	0.08	2.33	-2.55	1	-1.42	1.13	1.13	50 μg/kg/day				
49	c.1180C>T	Het	p.Arg394*	E7	IGD	Nonsense	N	SSOAOD		0					0	0	1	0	1	Barrel chest, broad forehead, limited supination, osteochondritis dissecans			–	–		-3.9									
50	c.1243G>T	Het	p.Glu415*	E7	IGD	Nonsense	N	SSOAOD																											
51	c.1243G>T	Het	p.Glu415*	E7	IGD	Nonsense	N	SSOAOD	3.58	1	89.1	13.3	146.1(−4.84 SDS)	157.6(−0.93 SDS)	0	1	1			Frontal bossing, macrocephaly, right eyelid ptosis, broad toes, retruded nose, micrognathia, mild pectus excavatum, flat chest	45.3	NA													
52	c.1308_1309del	Het	p.Gly437Argfs*22	E7	IGD	Frameshift	N	SSOAOD	4.58	0	96	18	175	146							132	8.72	5.5	0.92	4.5	-2.8	4	-1.3	0.375	1.5					
53	c.1411C>T	Het	p.Gln471*	E7	IGD	Nonsense	N	SSOAOD	4.67	0	92.8	13				1	1	1	0	Frontal compression, broad nasal bridge, elongated pharyngeal isthmus, internal rotation of the elbow	119		7	2.33		-3.48									
54	c.1425delA	Het	p.Val478Serfs*	E8	G2-B	Frameshift	N	SSOAOD	7.4	1															7.4	-2.9	3	-1.7	0.4	1.2	30-50 μg/kg/day				
55	c.1429+1G>C	Het	Val465Glyfs*13	E8	G2-8	Splicing	N	SSOAOD	7.4	0			173	143		NA	NA	NA	NA	Precocious puberty		8.59	8.8	1.4	8.83	-3.33	1.75	-2.17	0.662857143	1.16		0.75	10.3	1	
56	c.1429+1G>C	Het	Val465Glyfs*13	E8	G2-8	Splicing	N	SSOAOD	5.75	0	106.5	16.5	159	162	0						203	13.067	5.75		6	-2	1	-1.4	0.6	0.6	0.16IU/kg/d				
57	c.1429+1G>C	Het	Val465Glyfs*13	E8	G2-8	Splicing	N	SSOAOD	10.83	0	142.4	41	159	162	0								11.5-12												
58	c.1443G>T	Het	p.Glu415*	E7	G2-B	Nonsense	N	SSOAOD	8.5	0															8.5	-1.7	2.1	-0.5	0.571428571	1.2	30-50 μg/kg/day	1			
59	c.1443G>T	Het	p.Glu415*	E7	G2-B	Nonsense	N	SSOAOD	5.7	1															5.7	-1.7	0.5	-1.5	0.4	0.2	30-50 μg/kg/day				
60	c.1443G>T	Het	p.Glu415*	E7	G2-B	Nonsense	N	SSOAOD	8.4	0															8.4	-0.7	0.8	-0.5	0.3	0.2	30-50 μg/kg/day				
61	c.1443G>T	Het	p.Glu415*	E7	G2-B	Nonsense	N	SSOAOD	3.2	0															3.2	-3	0.8	-2.3	1.05	0.7	30-50 μg/kg/day				
62	c.1526C>A	Het	p.Ser509*	E8	G2-B	Nonsense	N	SSOAOD																											
63	c.1608C>A	Het	p.Tyr536*	E9	G2-B	Nonsense	N	SSOAOD	5	0	94.5			-4.7	1	1	1	0	1	Mild knee cartilage defect			4.5		5	-3.7	9	-3.9	-0.022222222	-0.2	1-2mg/m2/d	1.5	17	3	145.5(-3.0)
64	c.1608C>A	Het	p.Tyr536*	E9	G2-B	Nonsense	N	SSOAOD	11.9	1					1	1	1	0	1	Mild posterior rotation of the ears, broad great toes, osteoarthritis, left renal agenesis						-2.5	3.5	-1.6	0.257142857	0.9	2 mg/m2/d	2			
65	c.1702G>A	Het	p.Asp568Asn	E9	G2-B	Missense	N	SSOAOD		1							0	0	0	Barrel chest, limited supination			–	–		-3.2									
66	c.1733-1G>A	Het		I9		Splicing	N	SSOAOD	20.42	0	143.2	48			0	1	1			Elongated pharyngeal isthmus, epiphyseal closure						-3.24									
67	c.1744delT	Het	p.Phe582fs*69	E10	G2-B'	Frameshift	N	SSOAOD	10	1	121.2	38		141.5(-3.0)	0								11.5	1.5	11.5	-2.1	2	-2	0.05	0.1	0.175 ug/kg/day	1			
68	c.1762C>T	Het	p.Gln588*	Exon10	G2-B’	nonsense	N	SSOAOD	9.83	1	114.3	22.5			1	1	0	0	1	Broad nasal bridge, elongated pharyngeal isthmus, flared ribs	111		7.5	-2.33		-4.18									
69	c.1774C>T	Het	p.Gln592*	E10	G2-B'	Nonsense	N	SSOAOD		1							1		0	Barrel chest			–	–		-3.2									
70	c.1817C>A	Het	p.Ala606Asp	E10	G2-B'	Missense	N	SSOAOD	9.42	1	120.6	22			1	1	0	0	1	Broad nasal bridge, elongated pharyngeal isthmus, flared ribs, café-au-lait macules	17.4	4.63	8	-1.42	9.42	-2.88	2.5	-1.91	0.388	0.97					
71	c.1817C>A	Het	p.Ala606Asp	E10	G2-B'	Missense	N	SSOAOD	6.92	0	118.7	24	166	152	0						287	14.213	8.75	1.83	7	-0.7	1.5	-0.1	0.4	0.6	0.17IU/kg/d				
72	c.1817C>A	Het	p.Ala606Asp	E10	G2-B'	Missense	N	SSOAOD	2.75	1	87.4	13	166	152	0						NA	NA	3		2.92	-2.1	0.5	-1.4	1.4	0.7	0.12IU/kg/d				
73	c. 1877G>A	Het	p.W626	E10	G2-B'	Nonsense	N	SSOAOD	5.08	1	103.3	18	150 cm(-3.75 SD)	153 cm(-1.41 SD)							162	8.35	5.75	0.65	5.4	-2	1	-1.77	0.23	0.23	0.12 U/(kg·d)				
74	c.1888G > A	Het	p.G630S	E10	G2-B'	?	N	SSOAOD	5.67	1	102.4	16.5	170	157	1						63.4	2.58	5.5	-0.17		-3									
75	c.1930G>A	Het	p.Gly644Ser	E10	G2-B'	Missense	N	SSOAOD	16	0					0	0	0	0	1	Madelung deformity, short femoral neck, knee epiphyseal plate defect			16	0		-2.1									
76	c.1948G>A	Het	p.Val650Met	E10	G2-B'	Missense	N	SSOAOD	12	1					0	0	0	0	1	Epicanthal folds, thin lips, depressed nasal bridge			9	-3		-2.6									
77	c.1967A > G	Het	p.Y656C	E10	G2-B'	?	N	SSOAOD	5.08	0	95.2	12.7	167	150	0						96.8	10.06	5	-0.08		-3.6									
78	c.1979C>T	Het	p.Thr660Met	E10	G2-B'	Missense	NA	UVS (PP3 + BS2)	10.58	1			-1.34	0.93	1							21.3	10.5	-0.2		-2.1									
79	c.1979C > T	Het	p.Thr660Met	E10	G2-B'	Missense	NA	UVS (PP3 + BS2)	3.5	1	92.6	14	170	160	0						58.2	4.339	2-2.5	-1.25		-2.1									
80	c.2023C>T	Het	p.Arg675*	E10	G2-B'	Nonsense	N	SSOAOD	5.75	1	100.4	15			1	0	0	0	0	Mild hyperopia, broad nose, Flat nasal bridge		19.9	7.5	1.75	6	-3.8	1.5	-3	0.53	0.8	56.1-62.7ug/kg/day				
81	c.2023C>T	Het	p.Arg675*	E10	G2-B'	Nonsense	N	SSOAOD	3.5	1													5	1.5	3.5	-2.49	1	-1.1	1.39	1.39	50 μg/kg/day				
82	c.2023C>T	Het	p.Arg675*	E10	G2-B'	Nonsense	N	SSOAOD	9.58	0													8.67	-0.91	9.58	-3.48	1	-2.71	0.77	0.77	50 μg/kg/day				
83	c.2026+1G>A	Het		I10	G2-B'	Splicing	N	SSOAOD	14.08	0	138.2				0	1	0	1	1	Type 1 diabetes mellitus, mildly broad forehead, posteriorly rotated ears						-4									
84	c.2153C > A	Het	p.T718K	E11	KS	Missense	N	SSOAOD	6	1	103.1	17.5			0	0	0	0	1		151	7.39	6.67	0.67	6	-3.22	1.67	-1.37	1.107784431	1.85	0.15 IU/kg/day				
85	c.2164C>G	Het	p.Pro722Ala	E11	KS	Missense	N	SSOAOD	5.33	0	99.4		153(-3.3SDS)	169(+1.58SDS)	1	0	0	0	0	Central precocious puberty	267.8	9.53	6	0.17	5.33	-3.13	0.5	-2.08	0.7	1.05	0.1 ug/kg/day	1	6	-0.83	/
86	c.2173G > T	Het	p.Glu725*	E11	KS	Nonsense	N	SSOAOD	10.08	1	122.7	25.5	153	158	0						365	>34.8	10.5	0.42		-2.91									
87	c.2218A>T	Het	p.Thr740Ser	E11	KS	Missense	N	SSOAOD							0	1	0	0	1	Frontal compression, prominent palate, triangular face			3	0											
88	c.2266G>C	Het	p.Gly756Arg	E11	KS	Missense	N	SSOAOD	3.33	1	89.2	15			0	1	1	1		Frontal compression, broad nasal bridge, Elongated pharyngeal isthmus, Flared ribs	138	5.06	4	0.67	3.92	-2.92	0.92	-2.16	0.826086957	0.76					
89	c.2369C>G	Het	p.Ser790*	E12	KS	Nonsense	Y	SSOAOD	14.5	1					1	0	0	1	1	Frontal compression, broad nasal bridge, hypertelorism			16.5	2		-2.2									
90	c.2398delinsA	Het	p.Ser800Profs	E12	KS	Frameshift	NA	SSOAOD	1.33	0	71.3	8	150	160	0											-2.88									
91	c.2535_2536insTTCA	Het	p.Pro846Phefs*9	E12	KS	Frameshift	NA	SSOAOD	4.75	1			-0.48	0.17	0							7.4	4.5	-0.58		-2.47									
92	c.2660C > G	Het	p.S887X	E12	KS	Nonsense	N	SSOAOD	2.75	1	85.5	11			0						60	4.2	2	-0.75	2.75	-3.05	5.25	-1.35	0.32	1.7	0.15 IU/kg/day				
93	c.2831dupT	Het	p.Glu945Argfs*484	E12	CS1	Frameshift	NA	SSOAOD	6.58	0			153	146		0	1	1	1	Depressed nasal bridge, prominent forehead, cubitus valgus		16.8	10	3	7.1	-2	2.25	-1.47	0.235555556	0.53		2	11.7	2.2	
94	c.2911G > T	Het	p.G971X	E12	CS1	Nonsense	N	SSOAOD	3.33	1	89	13.5					1		1	Depressed nasal bridge	91		3.67	0.33	3.33	-3.08	0.25	−2.46	2.48	0.62	0.15 IU/kg/day				
95	c.3127_3642del	Het	p.Ala1043_Ala1214del	E12	CS1	Missense	NA	SSOAOD	8.5	0			163	153		0	0	0	0	Depressed nasal bridge, pectus carinatum, precocious puberty		25.9	9.83	0.3	9.7	-3	0.5	-2.42	1.16	0.58					
96	c.3587delC	Het	p.Pro1196Leufs*3	E12	CS1	Frameshift	N	SSOAOD	3.08	1	87.2	14	155	159	0								4	0.92		-2.7									
97	c.4657G>T	Het	p.Glu1553*	E12	CS1	Nonsense	N	SSOAOD	8.7	0															8.7	-2.9	0.3	-2.6	0.45	0.3	30-50 μg/kg/day				
98	c.4762_4765del	Het	p.Gly1588fs	E12	CS1	Frameshift	N	SSOAOD	12.25	1			-3.8	0.6	1	1	0	0	1	Posteriorly rotated ears				1	12.25	-2.7	6.2	-2.6	0.016129032		1mg/m2/d				165.3(-2.6)
99	c.4852C>T	Het	p.Gln1618*	E12	CS2	Nonsense	N	SSOAOD	11.83	1	120	23.5			0							9.3	11.83	0	11.83	-4	1	-3.8	0.2	0.2	0.29mg/kg/w				
100	c.5058_5059delCA	Het	p.Ile1686Metfs*13	E12	CS2	Frameshift	N	SSOAOD	12.92	1	137.9	46.3	158	163	0						530	6.219			12.75	-2.7	0.42	-2.6	0.238095238	0.1	0.16IU/kg/d				
101	c.5185delA	Het	p.lle1729Tyrfs*24	E12	CS	Frameshift	N	SSOAOD	8.8	0			154	146						Prominent forehead, low-set ears, depressed nasal bridge		9.27	10.2	1.5	8.92	-2.81	2.5	-2.27	0.216	0.54		2.5	12	0.5	
102	c.5391delG	Het	p.Gly1797Glyfs*52	E12	CS2	Frameshift	N	SSOAOD	5.5	1	99			147.7(-2.6)	0	1					128		8	2.5	6.17	-2.6	1	-2	0.6	0.6	30-50 μg/kg/day				
103	c.5597C>A	Het	p.Ser1866*	E12	CS2	Nonsense	Y	SSOAOD		1						0	1	0	1	Frontal compression, barrel chest			+	+		-2									
104	c.6142C>G	Het	p.Pro2048Ala	E12	CS2	Missense	N	SSOAOD	12.5	0					1	0	0	0	1	0			12.5	0		-2.2									
105	c.6176del	Het	p.Pro2059Leufs*2	E12	CS2	Frameshift	N	SSOAOD	6.5	1	104.5	17.5			1	0	1	1	0	Mild hyperopia, broad nose, long philtrum		10.2	7.5	1	6.5	-3.3	0.66	-3	0.45	0.3	56.1-62.7ug/kg/day				
106	c.6193delC	Het	p.Gln2065Serfs*27	E12	CS2	Frameshift	N	SSOAOD	9.83	1	115	25.4									308	5.89	9.83	0		-4									
107	c.6229delG	Het	p.Asp2078Thrfs*14	E12	CS2	Nonsense	y	SSOAOD	9.75	1	131.8		150(-3.74SD)												6	–	3.75				66 μg/kg/day				
108	c.6337delG	Het	p.Ala2113fs*17	E12	CS2	Frameshift		SSOAOD	7.8	0			166	144		1	0	1	0	Depressed nasal bridge		13.08	9.6	1.9	7.9	-2	0.5	-1.15	1.7	0.85			9.83	1.83	
109	c.6404delC	Het	p.Ala2135Aspfs	E12	CS2	Frameshift	Y	SSOAOD	6.75	1			-0.76	0.7																					
110	c.6665G > A	Het	p.Trp2222*	E12	CS2	Nonsense	N	SSOAOD	8.17	0	116.5	23	170	142	1						212	12.631													
111	c.6679C>T		p.Gln2227*	E12	CS2	Nonsense	Y		4.83	1	94	14.4	165(−1.67 SD)	130(−6.48 SD)	0											-3.34									
112	c.7038_7039insCGGTGT		p. Arg2349_Glu2 350insCysArg						5	1	101.2	17.6	170 cm (-0.42 SD)	145 cm (-2.89 SD)							99	8.9	5.5	0.5		-2.39	3	-0.6	0.596666667	1.79	0.12 U/(kg·d)				
113	c.7064T>C	Het	p.Leu2355Pro	E14	G3	Missense	N	SSOAOD	14.75	0	135.8					0			1	Relative macrocephaly (head circumference > 2 SDS), mild bilateral genu valgum, osteochondritis dissecans			13	1.42	12	-3.2	1.5	-3.9	-0.466666667	-0.7	30-50 μg/kg/day	1	13	-0.5	-3.9
114	c.7090C>T	Het	p.Gln2364*	E15	G3	Nonsense	N	SSOAOD	11.58	1			-2	-4.9		1	0	1	1	Osteochondritis dissecans			12.25	0.8	11.58	-2.7	5.6	-2.9	-0.035714286	-0.2	2mg/m2/d	2		1.25	
115	c.7091A>C	Het	p.Gln2364Pro	E15	G3	Missense	N	SSOAOD																											
116	c.7093_7095delGAG	Het	p.Glu2365del	E15	G3	Deletion	NA	SSOAOD	8.8	1			-1.85	-0.59	0							9.1	7.5	-1.3		-3.71									
117	c.7153G>A	Het	p.Glu2385Lys	E15	G3	Missense	N	SSOAOD																											
118	c.7153G>A	Het	p.Glu2385Lys	E15	G3	Missense	N	SSOAOD	5.5	1			170	149	NA	0	0	0	0	Depressed nasal bridge, intellectual disability		25.5													
119	c.7198G > T	Het	p.Glu2400*	E15	G3	Nonsense	N	SSOAOD	3	0	87.3	12.3	162	145	1						110		4.2	1.2		-2.24									
120	c.7202G>A	Het	p.Trp2401*	E16	G3	Nonsense	N	SSOAOD	5.17	0													6.67	1.5	5.17	-1.07	1	-0.72	0.35	0.35	50 μg/kg/day				
121	c.7202G>A	Het	p.Trp2401*	E16	G3	Nonsense	N	SSOAOD	6.75	0													7.67	0.92	6.75	-2.26	1	-1.71	0.55	0.55	50 μg/kg/day				
122	c.7203G>A	Het	p.Trp2401*	E16	G3	Nonsense	N	SSOAOD																											
123	c.7211_7214del	Het	p.Asn2404Serfs*14	E16	G3	Frameshift	Y	SSOAOD	3.08	1			172	149	1	1	1	1	0	Prominent forehead, depressed nasal bridge, low-set ears		4.64	4.4	0.93	3.5	-3.13	2	-2.07	0.53	1.06			6.8	1.3	
124	c.7222dupA	Het	p.Asp2407fs	E16	G3	Frameshift	NA	SSOAOD	11	0			170	141						Menarche at 12 years of age	521	10		0.4		-2.36									
125	c.7243delG	Het	p.D2415Tfs*4	E16	G3	Frameshift	N	SSOAOD	9.33	1	125	35.7			0	0	1	0	1		205	2.33	10		9.33	-2.18	2.75	−0.44	0.63	1.74	0.15 IU/kg/day				
126	c.7269delG	Het	p.Glu2424fs*5	E16	G3	Frameshift	N	SSOAOD	1.5	1					0	0	0	0	1	Depressed nasal bridge			3.5	2		-3									
127	c.7276G>A	Het	p.Glu2426Lys	E16	G3	Missense	N	SSOAOD	8.5	0					1	0	0	0	1	Depressed nasal bridge, frontal compression, conical epiphyses			8.5	0		-2.5									
128	c.7276G>T	Het	p.Gly2426*	E16	G3	Nonsense	N	SSOAOD	18	0					1	0	0	0	1	Macrocephaly, short femoral neck			18	0		-4.3									
129	c.7276G>A	Het	p.Glu2426Lys	E16	G3	Missense	N	SSOAOD	7.5	0			166	155		1			1	Depressed nasal bridge		11.1	6.8	-1.2		-2.52	0.75	-1.85	0.893333333	0.67					
130	c.7276G>A	Het	p.Glu2426Lys	E16	G3	Missense	N	SSOAOD	9.25	0			160	157		1	0	1	0	Depressed nasal bridge, precocious puberty		16.6	7.8	-1.9	9.5	-2.06	0.5	-1.87	0.38	0.19					
131	c.7293del	Het	p.Cys2432Alafs*139	E16	CLD	Frameshift	N	SSOAOD	8	0	111				NA	0	0	0	1	0						-3.2									
132	c.7342G>A	Het	p.Gly2448Arg	E17	G3	Missense	NA	SSOAOD	46	0					NA	1	0	0	1	Frontal compression			NA	NA		-3.7									
133	c.7360_7361del	Het	p.E24 54fs	E17	G3	Frameshift	NA	SSOAOD	4.08	0	94	14.6									90	12.1	4	-0.1		-2.52	0.5	-2.05	0.94	0.47	0.13 U/(kg·d)				
134	c.7429G>A	Het	p.Val2417Met	E16	G3	Missense	N	SSOAOD																	5.5	-1.2	3.8	-0.7	0.131578947		30-50 μg/kg/day				
135	c.7429G>A	Het	p.Val2417Met	E16	G3	Missense	N	SSOAOD																		-0.8	1.1	-0.2	0.545454545	0.6	30-50 μg/kg/day	1			
136	c.7465C > G	Het	p.Arg2489Gly	E17	G3	Missense	N	SSOAOD	7.25	1	114.8	26	157	152	0						135	8.46	7.5	0.08	7.42	-2.1	0.75	-1.7	0.533333333	0.4	0.13IU/kg/d	1			
137	c.7465T>C	Het	p.Gln2364Pro	E17	G3	Missense	NA	SSOAOD	5.5	0	104.5	18			0						290	12.1	6.5	0.92		-2.09	3.9	-1.07	0.26		40 μg/kg/day	2			
138	c.7465T>C	Het	p.Gln2364Pro	E17	G3	Missense	NA	SSOAOD	7.67	1	122.5	25			0						200	NA				-0.88	5	-0.31	0.11		50 μg/kg/day	1			
139	c.7465T>C	Het	p.Gln2364Pro	E17	G3	Missense	NA	SSOAOD	6.33	1	100.5	17.5			0						148	15.1				-0.74	7.7	-3.75	0		60 μg/kg/d				
140	c.7469G>A	Het	p.Cys2490Tyr	E18	G3	Missense	N	SSOAOD	14.58	1	137.3	44.5			0	0	1	1	1	Pectus carinatum	399	8.13	12	-2.58	15.08		0.16	-4.37	1	0.16		1			-4.37

Analysis of 65 children treated with GH therapy demonstrated that median height SDS before treatment was -2.7 SD, which improved to -2.0 SD after treatment, with an average SDS improvement of 0.59 SD (2, 3, 25–27). The first year of treatment showed particularly significant effects (average SDS increase of 0.68 SD), indicating that GH has a sustained growth-promoting effect in most children and can improve final adult height to some extent. However, the efficacy was negatively correlated with age: the annual SDS increase reached 0.95 SD in children under 3 years old, while it decreased to approximately 0.38 SD in children over 9 years old. Additionally, combined treatment with GnRHa did not significantly enhance the efficacy (average SDS improvement of only 0.37 SD). Overall, GH therapy holds certain value in the management of SSOAOD, but there is still a lack of clear association between genotype and efficacy, and no targeted therapies are available (2, 3, 46, 48, 49). Further exploration of more precise treatment strategies and early intervention plans is required.

### 6.2 Bone and joint management

For patients with SSOAOD caused by heterozygous mutations in the *ACAN* gene, in addition to height management, it is crucial to systematically manage their associated skeletal complications such as osteoarthritis (OA) and osteochondritis dissecans (OCD). After entering adulthood, for progressive and severe OA or OCD, orthopedic intervention may be required. Measures may include arthroscopic debridement, cartilage repair techniques, or joint replacement in the end-stage to alleviate pain and improve joint function (15). Surgery is a symptomatic treatment for OCD complications (loose bodies) and end-stage osteoarthritis, aiming to relieve pain and improve function without altering the fundamental progression of the disease. Some patients had recurrence of symptoms or still needed further arthroplasty. There is a lack of long-term follow-up and multidisciplinary management consensus (1).

### 6.3 Follow-up and genetic counseling

For patients with SSOAOD caused by heterozygous mutations in the *ACAN* gene and their families, systematic follow-up and genetic counseling are essential for management. First, given its autosomal dominant inheritance pattern, genetic screening of first-degree relatives of the proband is crucial, which facilitates early identification of asymptomatic or mild cases, enabling early diagnosis and intervention within the family. Secondly, a multidisciplinary expectant management and long-term follow-up system should be established, led by the endocrinology department and involving the orthopedics/rehabilitation departments. This system should regularly monitor the height, bone age, scoliosis, and joint symptoms (pain, limited mobility) of confirmed individuals, and evaluate the efficacy and safety of growth hormone (GH) treatment. For those with existing joint lesions, individualized exercise recommendations and imaging follow-up plans should be developed. The core of genetic counseling lies in elucidating the inheritance pattern, penetrance, and phenotypic variability of the disease to the family, providing recurrence risk assessment, and discussing reproductive options such as prenatal diagnosis to assist the family in making informed decisions (2, 3, 12, 15).

## 7 Research frontiers and unsolved problems

Regarding SSOAOD caused by heterozygous mutations in the *ACAN* gene, current frontier research primarily focus on: First, the precise molecular mechanism of its core pathogenic pattern, *ACAN* haploinsufficiency, has not been fully elucidated. How mutations in different domains differently affect protein stability, secretion, and extracellular matrix assembly, thereby driving growth plate failure and joint degeneration, still requires further exploration (7, 10). Secondly, although growth hormone (GH) is the primary treatment, its long-term safety impact on the already fragile articular cartilage of patients remains unclear, and there is a lack of long-term follow-up data to assess whether it accelerates the progression of osteoarthritis (2, 3). Furthermore, apart from the known GH-IGF1 and chondrocyte-derived osteogenic signaling pathways, whether other metabolic or inflammatory pathways are involved in phenotypic regulation, particularly in the pathogenesis of early-onset arthritis, represents a promising new research direction (15). Finally, due to the high variability of phenotypes and the lack of specific biomarkers, there is no unified clinical and biochemical standard for early childhood identification. The key challenge in improving diagnostic rates and enabling early intervention lies in how to accurately and cost-effectively screen for *ACAN* mutations among numerous children with short stature (12, 15).

## 8 Conclusion

The heterozygous mutation of the *ACAN* gene is one of the important genetic bases for non-syndromic familial short stature and early degeneration of bones and joints, resulting in a spectrum of SSOAOD diseases that exhibits a continuous pathological process from growth plate dysfunction (short stature, abnormal bone age) to premature degeneration of articular cartilage (early-onset OA/OCD). Currently, rhGH is the primary means to improve height of patients, but its efficacy is age-dependent, with unclear long-term joint. Clinical management should integrate growth promotion, management of skeletal complications and genetic counseling.

Looking ahead, the key to overcoming current knowledge gaps lies in establishing large-scale, multicenter prospective cohorts with long-term follow-up, which will help: 1) elucidate the precise molecular mechanisms and phenotypic associations of *ACAN* haploinsufficiency; 2) clarify the long-term benefit-risk profile of GH therapy, particularly its effects on joints; 3) develop unified criteria for early identification in childhood; 4) ultimately lay a solid clinical and research foundation for the development of targeted therapies (e.g., signaling pathway modulators or gene therapies).

## Data Availability

The datasets presented in this study can be found in online repositories. The names of the repository/repositories and accession number(s) can be found in the article.
